# Supplementary material for: Repeated automatic sleep scoring based on ear-EEG is a valuable alternative to manually scored polysomnography
Source: PLOS Digit Health. 2022 Oct 27;1(10):e0000134. doi: 10.1371/journal.pdig.0000134 (PMC9931275; doi:10.1371/journal.pdig.0000134)
Supplement: Attachment — Submitted filename: reviewer_response.rtf [file pdig.0000134.s001.rtf]

Reviewer #1: GENERAL COMMENTS
Dear authors,
I appreciated the reading of your article.
However, I have several concerns about the study setting, some missing details and the reliability of the results, because, if I correctly understand, the authors compared four nights of the wearable device with one night of PSG.
Please see the following comments

INTRODUCTION
1.      Sleep represents an issue also in cancer. I suggest adding the following article: Castelli et al., (2021), DOI: 10.1007/s00520-021-06377-5.
This has now been incorporated

2.      In my opinion, respiratory disease instead of respiratory issues is more appropriate.
We have now added 'respiratory disorder' as well.

3.      Please pay attention that the cited articles are not correctly reported in the text. Furthermore, they are reported in different formats.
We have now made sure that all citations are on the format (first-author et al., year). If there are less than three authors, all names are given. We have ensured that all articles in the bibliography are in fact cited in the text.

4.      Please check that all the cited articles are present in the reference list.
We have now ensured this.

5.      The authors say only wearable. What does this mean? Which kind of device. Maybe, in the introduction, there should be more information to introduce the reader to the device and its advantages regarding PSG.
We have added the following to the introduction:
“..... wearable, more precisely the ear-EEG (Mikkelsen et al 2015). The ear-EEG is a promising mobile sleep monitor, which has proven to be both reliable and accurate (Mikkelsen et al. 2021).”
And:
“The promise of this approach is that given a good mobile sleep monitor, multiple wearable nights are likely to be cheaper and more practical to obtain than a single PSG night. Thus, our aim is to quantify at what amount of data wearable sleep monitoring (in this case ear-EEG) is superior to single PSG nights.”

6.      The aim is not precise; the authors just reported the tested hypothesis.
See above.

MATERIALS AND METHODS
1.      Lots of information are missing about participants, and the protocol is not clear: where and how have they been recruited? Which were the inclusion and exclusion criteria? Where did they sleep for the assessments night?
As was written in the earlier version of the manuscript, an exhaustive description of the data set was given in Mikkelsen et al 2019. However, to comply the request of the reviewer and make this manuscript more independent, the following has been added to the methods section:
“The participants were recruited primarily among the healthy student population through word of mouth. Exclusion criteria were: known sleep issues, contact-allergies to the materials, risk of sleep apnea, psychiatric disorders, sleep affecting medications and pregnancy. All recordings took place in the subject's own home.”

2.      Did the participants also use the PSG? Otherwise, how did the authors obtain the PSG data for the comparisons?
Yes, as stated in the methods section: 
“Twenty healthy volunteers were included after verbal and written informed consent and equipped with simultaneously recording PSG and ear-EEG equipment for four nights each.”

3.      Where did the evaluation take place?
See above

4.      The model, the characteristics, the manufacturer and other important details about the device are not reported.
The hardware was developed in-house. We have added to the manuscript that production details can be found in the original 2019 paper.
5.      Who elaborated the computer algorithm?
	
We have added the following: 
“The algorithm was identical to the one presented in Mikkelsen et al. 2019, implemented in MATLAB.”
6.      How did the authors evaluated and choose the two sleep technologists?

The two sleep technologists were not chosen due to any specific criteria, but they were both highly trained, working in different sleep clinics. We have added the following:
“The two sleep technologists did not train together, and were affiliated with different sleep clinics.”
7.      In my opinion, the authors should report how many epochs they discarded, because this could have influenced the goodness of the data.
This is described in detail in Mikkelsen et al 2019. We have now chosen to highlight this to any interested readers.

8.      Why did the authors decide not to evaluate the N1 fraction of sleep?
We thought 8 different statistics was a suitable number, and because the N1 sleep stage is notoriously hard to score for both people and machines, making that particular comparison very noisy.

9.      Statistical analysis is not clear, and it is not easy to follow the exposition of the results without a more precise explanation:
1.      What does “j nights” mean?
In this context, 'j' is an integer. As described in first usage, it is an integer from 1 to 4. This is the standard way of using mathematical variables in text. To perhaps make things clearer, we have changed 'j' to 'M'.

2.      Which statistical software has been used?
Matlab

3.      Is it possible to explain better the confusions matrices?
We believe the explanation given in the figure caption is already quite good. However, to make it easier for readers to find this, we have now included a mention indicating that an explanation is found in the caption.

4.      Which is the post-hoc used later in the results section?
In this case, post-hoc refers to the fact that we performed an analysis specifically to explain the results of a previous analysis (rather than something motivated purely from the research hypothesis). Specifically, we noticed that S1 and S2 disagreed on their N2 and N3 fractions, and digging into this, it was discovered that one scorer often transitioned from N2 to N3 later than the other scorer. As we only did this detailed analysis for this discrepancy, and not for all other types of transitions, we thought it prudent to label this as a 'post-hoc' analysis. 

5.      In general, lots of details are reported below the figures and not in the text; in my opinion, the authors should evaluate to integrate the information below the figures in the text.
Much of the discussion in the caption for figure 5 has now been moved into the text, in the place where figure 5 is mentioned. The rest of the details referred are mostly there to help the viewer interpret the given figure. As such, we believe it is useful to have it in the caption.

RESULTS
1.      “ in the range 0.7 to 0.74, indicating a substantial agreement”. Does this result mean that without a sleep technologist, the data could be interpreted correctly?
Yes, it means that the automatic scoring almost gives the same result as the manual scoring.

2.      In figure $, I suggest adding A, B, C in each panel to make it more understandable. It is not clear to which panel the authors refer.
Presumably the reviewer is referring to figure 6. We have now made the A,B,C clearer.

3.      I am wondering if it is correct comparing one PSG night to 4 wearable nights. Probably this is the intent of the authors and the article, but the wearable device is not precise in evaluating only one night, and it becomes more reliable after four nights. Did the authors test more nights? For example, one week? Why did they focus on four nights?
Yes, indeed, it was our intent to compare the reliability of a single PSG night to that of multiple ear-EEG nights. Given that the cost of multiple wearable nights is much lower than the cost of multiple PSG nights, it is valid to compare multiple wearable nights to a single PSG night. We find in this study that physicians are likely to get better data for their money by recording multiple wearable nights than a single PSG night, which is what is presently done in many places. In our rewriting of the discussion (as requested below), we have striven to make this clearer. 
We focused on four nights because that was how many nights were in the data set to begin with. To have extended the analysis to more nights, and been as rigorous as we are now, we would have had to also do PSG recordings on the same nights. That would have made the study significantly more expensive to perform.

4.      Table 1 is not recalled in the text.
See sentence two in the 'Methods' section.

DISCUSSION
1.      The authors should try to write the discussion more fluently; it appears as many paragraphs not connected one each other.
We agree. The discussion has now received a thorough rewriting.

2.      “We have worked extensively…” I do not understand the meaning of this paragraph.
We hope this is clearer in the reworked version.

3.      The authors should also consider the insertion of some consideration about the use of actigraph.
We have previously performed a detailed comparison of ear-EEG and actigraphy (using the same data set in fact), and found that ear-EEG was significantly better than a wrist-worn actigraph. This has now been pointed out in the discussion.

4.      Are these first results or first evidence or what else referring to this device? Are these data really usable from other research groups since the sample size is limited, in a healthy status and with a restricted age?
As should now be clear from the new version of the manuscript, we have published multiple papers about ear-EEG previously. A couple more are also on the way describing, describing large studies. However, it should be pointed out that when considering other validation studies of wearable EEG sleep monitoring, 80 nights is not a small number - it is quite expensive to perform these studies. Additionally, the AASM manual defines sleep stages based on 'normal' sleep EEG. As such, healthy sleep is the least biased domain in which to test new sleep technology such as mobile sleep monitoring devices. Finally, we are indeed in the process of performing new studies on patient populations, however, we could not do that before we had tested it on a healthy cohort.

5.      Maybe, the authors should describe a way for making this article more indispensable or useful for future studies.

We have added this to the discussion as well.
