# Supplementary material for: Repeated automatic sleep scoring based on ear-EEG is a valuable alternative to manually scored polysomnography
Source: PLOS Digit Health. 2022 Oct 27;1(10):e0000134. doi: 10.1371/journal.pdig.0000134 (PMC9931275; doi:10.1371/journal.pdig.0000134)
Supplement: Attachment — Submitted filename: reviewer response.docx [file pdig.0000134.s002.docx]

1. Please write the AASM abbreviation in full.

This has been done now.

2. 2.      In my opinion, you should write why you decided not to evaluate N1.

This has been added.

3. In my opinion, the fact that the automatic and the manual soring could be considered superimposable should be highlighted in the manuscript. Moreover, does this result means that ear-EEG recording could be performed at home or is the hospitalization still necessary?

Indeed, recordings can be performed at home, without outside assistance. We have published a conference paper about this recently, which has now been added to the manuscript (Mikkelsen et al 2022). We have added a single sentence highlighting that single-night performance is really quite close to the PSG device, in the discussion.
